# Personalizing Androgen Suppression for Prostate Cancer Using Mathematical Modeling

**DOI:** 10.1038/s41598-018-20788-1

**Published:** 2018-02-08

**Authors:** Yoshito Hirata, Kai Morino, Koichiro Akakura, Celestia S. Higano, Kazuyuki Aihara

**Affiliations:** 1 Institute of Industrial Science, The University of Tokyo, 4-6-1 Komaba, Meguro-ku, Tokyo, 153-8505 Japan; 2 Department of Mathematical Informatics, The University of Tokyo, Tokyo, Japan; 3 Department of Urology, JCHO Tokyo Shinjuku Medical Center, Japan Community Health Care Organization, Tokyo, Japan; 4 Department of Medicine, University of Washington and Fred Hutchinson Cancer Research Center, Seattle, Washington, USA

## Abstract

Using a dataset of 150 patients treated with intermittent androgen suppression (IAS) through a fixed treatment schedule, we retrospectively designed a personalized treatment schedule mathematically for each patient. We estimated 100 sets of parameter values for each patient by randomly resampling each patient’s time points to take into account the uncertainty for observations of prostate specific antigen (PSA). Then, we identified 3 types and classified patients accordingly: in type (i), the relapse, namely the divergence of PSA, can be prevented by IAS; in type (ii), the relapse can be delayed by IAS later than by continuous androgen suppression (CAS); in type (iii) IAS was not beneficial and therefore CAS would have been more appropriate in the long run. Moreover, we obtained a treatment schedule of hormone therapy by minimizing the PSA of 3 years later in the worst case scenario among the 100 parameter sets by searching exhaustively all over the possible treatment schedules. If the most frequent type among 100 sets was type (i), the maximal PSA tended to be kept less than 100 ng/ml longer in IAS than in CAS, while there was no statistical difference for the other cases. Thus, mathematically personalized IAS should be studied prospectively.

## Introduction

Intermittent androgen suppression (IAS) was proposed to hopefully overcome the relapse of prostate cancer that appears after long prescription of hormone therapy. In past clinical trials^1–14^ of IAS, after starting the hormone therapy, the therapy is stopped when the value of prostate specific antigen (PSA), a serum tumor marker in blood for prostate cancer, decreases to less than a predefined lower threshold value and the period of the hormone therapy becomes 6 or 9 months or above. The hormone therapy is resumed after PSA reaches an upper threshold value. The on- and off-treatment periods are alternated until the development of castration resistance. The same treatment schedule is applied to all patients uniformly.

We proposed a mathematical model of prostate cancer^15–19^ that describes the behavior of PSA under IAS quantitatively. Mathematically, the challenge is how to treat the uncertainty due to the short and noisy observations of PSA compared to time series data used in the other fields such as weather, renewable energy, and finance. In order to address this challenge, we have developed a method for overcoming this uncertainty using a statistical method called bootstrapping^20,21^. Thus, here we apply this statistical method to the datasets of 150 patients and examine what would have been an optimal treatment schedule of IAS for these patients using the mathematical model of Hirata *et al*.^15^.

## Materials and Methods

### Patient Data Analyzed

We analyzed 150 patients of prostate cancer treated by IAS. Out of 150 patients, 58 patients were the patients from the phase 2 study in Canada^8,12^, 17 were taken from Japan, and 75 were taken from the United States^22–25^. The analysis of the Canadian phase 2 study was approved by the ethics committee at the University of Tokyo School of Medicine (Review No. 2857-(8)). This Canadian study is too old to have the registry name or registration number of this trial. This study was previously analyzed in refs^8,12,15–18,26^. The Japanese cases were approved by the ethics committees of the JCHO Tokyo Shinjuku Medical Center (no approval number assigned) and The University of Tokyo (Review No. 2857-(8)). Each patient provided his oral informed consent. Prior to the oral informed consent, the ethics committee of JCHO Tokyo Shinjuku Medical Center approved that the oral consent is sufficient because the Japanese cases were part of usual clinical practice, retrospective, and did not have any intervention. The medical doctor for each patient recorded the patient’s oral consent in the medical record. These Japanese cases were previously analyzed in refs^18,26^. The American study was the on-going phase 2 study of IAS^22–25^. The NCI number is NCT00223665. The University of Washington (Protocol # 97-3730-A) and The University of Tokyo (Review No. 2857-(8)) approved this American study. All patients provided written informed consent. This American study was previously analyzed in^22–26^. All methods were performed in accordance with the relevant guidelines and regulations.

### Quantitative Mathematical Model of Hormonal Therapy

**Figure 1:**
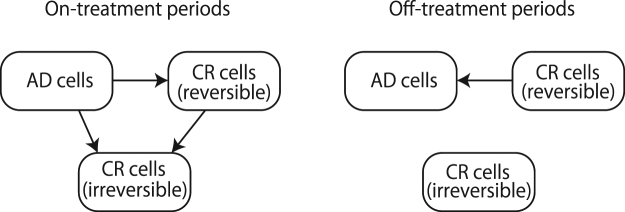
Schematic diagram for our mathematical model. AD cells correspond to androgen dependent cancer cells and CR cells correspond to castration resistant cancer cells.

The mathematical model we used here is an extension^15–19^ of model proposed in Ideta *et al*.^27^ (see Appendix A.1). This mathematical model consists of three variables: one kind of androgen dependent (AD) cancer cells, and two kinds of castration resistant (CR) cancer cells which are generated from AD cancer cells through reversible and irreversible changes, respectively. During an on-treatment period, AD cells may change to CR cells (see Fig. 1). During an off-treatment period, CR cells generated through reversible changes may become AD cells again. However, CR cells generated through irreversible changes such as mutations cannot change back to AD cells (see Fig. 1). By enforcing some constraints during fitting, this mathematical model can reproduce the relapse of cancer as well^15,18^.

### Fitting The Mathematical Model

**Figure 2:**
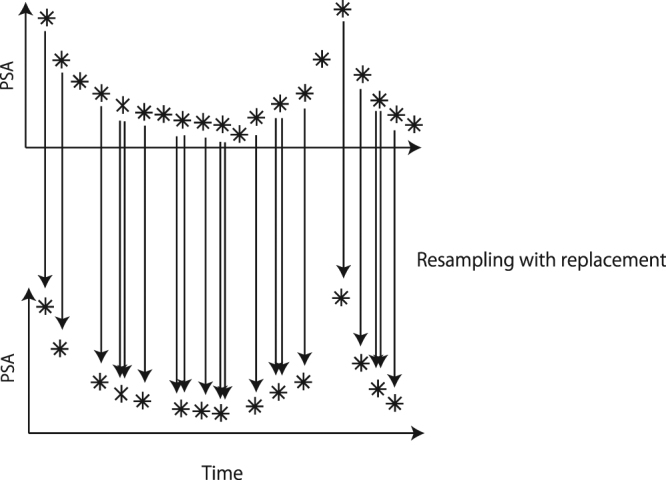
Schematic diagram for bootstrapping method. We resample data points with replacement. If two arrows are drawn, it means that this time point has been selected twice.

We first generated 100 bootstrap samples from PSA measurements obtained for each patient during the first 2 and half IAS according to Kuramae *et al*.^21^ (see Fig. 2 for the schematic illustration; see also Appendix A.2 for the details). Second, we fitted each bootstrap sample with the method proposed in^15^. The mathematical model can be fitted to the clinical datasets relatively well. Our method using the bootstrapping has been examined previously in Kuramae *et al*.^21^. Especially, in type (ii) and type (iii) patients, we found that the most frequent types chosen among the 100 bootstrap samples were also type (ii) and type (iii), respectively.

### Classifications of Patients

**Figure 3:**
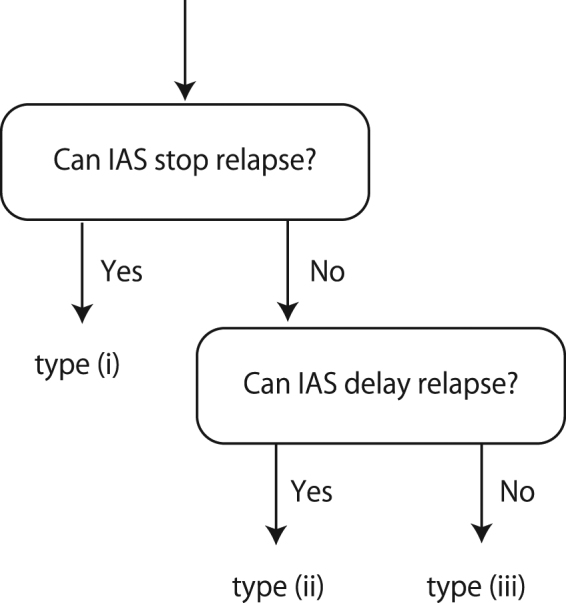
Classification of patients.

For each bootstrap sample, we examined which type the obtained parameter values corresponded to: type (i), where IAS stops the relapse and thus we target a periodic orbit by IAS^19,27^, type (ii), where IAS delays the relapse later than CAS and thus we try to delay the relapse of cancer by minimizing the growth rate of cancer, or type (iii), where CAS is better than IAS (see Fig. 3). See, the detail of the criteria in Appendix A.3 as well as^18^. We compared the classification made by the mathematical model with the initial Gleason score^28^, a pathological staging for prostate cancer by a biopsy, and the clinical status (without relapse, with metastasis, or with castration resistance) at the end of record we have.

### Optimal IAS

We minimized the PSA of 3 years later in the worst case scenario among the simulations of the estimated parameters whose simulation error in the PSA value was 5 ng/ml or less at the end of the first two and half cycles, out of 100 parameter sets for each patient. We searched exhaustively all over the possible schedules among such simulations where each hormone treatment is assigned as a block of 24 weeks and obtained an optimal treatment schedule for each patient. Since we searched exhaustively all over the possible schedules described above, our best solutions are not sub-optimal.

## Results

There were 150 prostate cancer patients whose Gleason scores were more than or equal to 6, treated with IAS: 58 from a phase 2 study in Canada^8,12^, 17 from Japan, and 75 from a phase 2 study in the United States^22–25^. The mean and the standard deviation for the number of time points among the patients were 33.7 ± 17.1, respectively. The minimum and the maximum for the number of time points were 4 and 103. The PSA values ranged between 0 and 220.00 (ng/ml). The mean and the standard deviation for the PSA values were 2.63 ± 6.57 (ng/ml), respectively. There were no pre-treatment PSA measurements.

**Figure 4:**
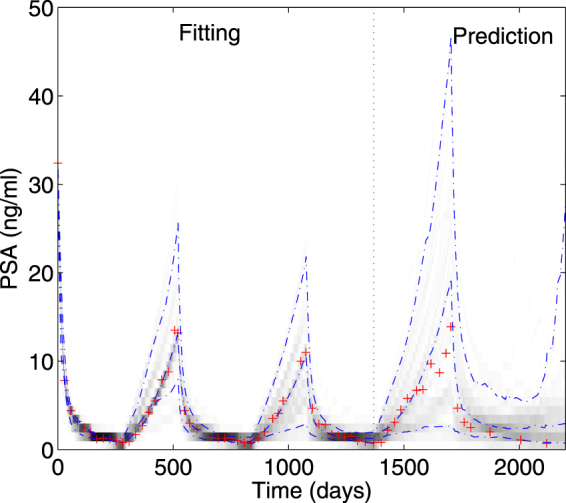
Fitting a mathematical model of prostate cancer to PSA values. Predictions of PSA follow the fitting period of the first 2 and half cycles of intermittent androgen suppression. Dash-dotted lines represent 10%, 50%, and 90% points of bootstrap samples for each time point. Crosses show actual measurements of PSA. The darkness shows the density of bootstrapped solutions.

**Figure 5:**
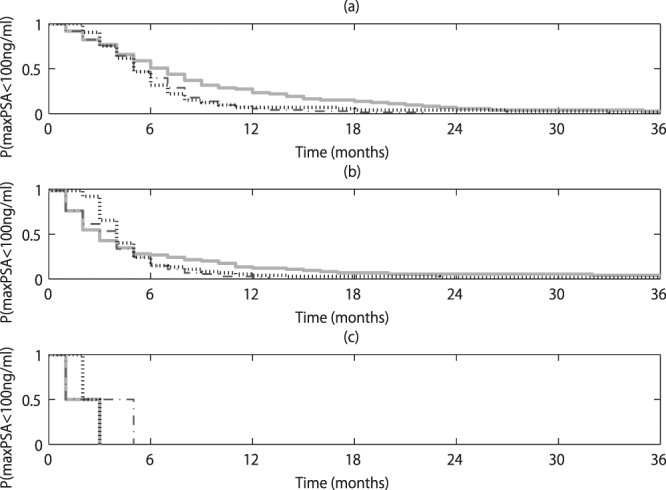
Time vs probability that the maximum PSA among 100 bootstrap samples is less than 100 ng/ml. The length of the time period that the maximal PSA value among 100 bootstrap samples is less than 100 ng/ml after the first 2 and half cycles was estimated. If patients were classified off-study before the first 2 and half cycles, then the time from the off study was estimated. Panel (a) is for type (i) patients, panel (b) is for type (ii) patients, and panel (c) is for type (iii) patients. In each panel, the solid line corresponds to the optimized IAS, the dash-dotted line corresponds to the conventional IAS, and the dotted line corresponds to CAS. In the conventional IAS, the hormone therapy is stopped when the PSA level is less than 0.1 ng/ml 40 weeks after the restart of the hormone therapy and the hormone therapy is resumed when the PSA level becomes greater than or equal to 1 ng/ml.

By fitting the first 2 and half cycles of IAS, one can predict future behavior with the precision shown in Fig. 4 (see Fig. 5 of^15^ for the validity for fitting single PSA time series). Therefore, the mathematical model of^15^ provides quantitative prognosis of prostate cancer. Examples for the estimated parameters for type (i), type (ii), and type (iii) are shown in Supplementary Tables 1, 2 and 3, respectively.

The mathematical model can distinguish patients without relapse from patients with metastasis and those with castration resistance. Table 1 shows the results of the comparison of mathematical model types (i–iii) with clinical status at the end of record. We found that if the clinical status was “without the relapse”, the most frequent type among 100 bootstrap samples was likely to be type (i). For patients classified with metastasis or with castration resistance, the possibility that they were either type (ii) or type (iii) is greater (the odds ratio: 2.80, P = 0.0029 (using the Fisher’s exact test in R)). This correlation implies that if a patient is classified to type (ii) or type (iii), the patient has a higher possibility to have metastasis or castration resistance.

**Table 1:** Classification by the mathematical model in relation to clinical status at the end of record.

Mathematical Model types	End of record			Total
	Without relapse	With metastasis	Castration resistance	
Type (i)	52 (71%)	9 (12%)	12 (16%)	73 (100%)
Type (ii)	36 (48%)	9 (12%)	30 (40%)	75 (100%)
Type (iii)	0 (0%)	1 (50%)	1 (50%)	2 (100%)
Total	88 (59%)	17 (13%)	43 (29%)	150 (100%)

We also compared the Gleason score^28^ with the most frequent type (i), (ii), or (iii) among 100 bootstrap samples obtained by the fitting. But we could not find significant correlation between the Gleason score and the most frequent type of patients (Table 2; P:0.13).

**Table 2:** Classification by mathematical model related to Gleason score, pathological stages for prostate cancer.

Mathematical model Types	Gleason score		Total
	≤8	≥9	
Type (i)	70 (96%)	3 (4%)	73 (100%)
Type (ii)	65 (87%)	10 (13%)	75 (100%)
Type (iii)	2 (100%)	0 (0%)	2 (100%)
Total	137 (91%)	13 (9%)	150 (100%)

Actually, by personally optimizing schedules, we found that IAS is more preferred to CAS (Fig. 5). Especially, when the most frequent type among 100 bootstrap samples was type (i), IAS tended to keep the PSA level for the worst case scenario among the bootstrap sample simulations less than 100 ng/ml for a longer time period than CAS (see Fig. 5a; P:0.024). In addition, while the average length of worst case survival with the PSA less than 100 ng/ml among the bootstrap sample simulations for type (ii) patients was longer for IAS (6.1 months) than CAS (5.3 months), there was no statistical difference between IAS and CAS when the most frequent type was either type (ii) or type (iii) (see Fig. 5b and c; P = 0.40 and P = 0.50, respectively). The optimal schedules for type (i) patients vary, depending on the maximal growth rates among the cells as shown in Supplementary Fig. 1. On the other hand, the conventional IAS is inferior to CAS in terms of the time length for keeping the maximum PSA level less than 100 ng/ml for type (ii) patients (P = 0.027) while there were no statistically significant differences for type (i) patients (P = 0.33) and type (iii) patients (P = 0.80).

## Discussion

While the treatment schemata for IAS have been fixed on clinical trials, this retrospective analysis of 150 patients who were treated with the conventional IAS suggests that a more personalized approach may be feasible. Application of this mathematical model shows that the behavior of the prostate cancer can be predicted and used to decide when to reinstitute AS or whether to use CAS instead.

Many mathematical models have been proposed for IAS^15–19,27,29–31^. By considering several models simultaneously, we may be able to derive more robust treatment to control the prostate cancer. This direction is a topic for the future research.

When we averaged all the bootstrap samples and applied the mean dynamics starting from the mean state at the first two and half cycles among the simulations within which the simulation error in the PSA value there was 5 ng/ml or smaller, its optimized schedule was too optimistic, always reached the considered maximum length of 36 months for each patient, and was longer (96.7%) or equal (2.3%) than the corresponding optimized schedule shown in Fig. 5, which took into account each of the bootstrap samples. Therefore, if we use the mean dynamics only, we cannot consider the variety of the bootstrap samples, which describe the uncertainty in terms of the underlying dynamics finely.

The solutions we evaluated are based on the best schedules for IAS because they minimized the PSA level for the worst case scenario, or the maximal PSA level, among the estimated parameter sets corresponding to 100 bootstrap samples for each patient. A control scheme is defined as robust^32^ if a set of parameters has some uncertainty within a mathematical model and the control scheme attains an intended state even for the worst case scenario among the possible parameter sets. In order to optimize IAS in clinical practice, we need to prepare a control/optimization method that can be applied with prediction or fitting. Currently, we have the control/optimization approaches with model predictive control^33,34^ and minimization of the maximal growth rate^35^, both of which have not been within the framework of robust control, and thus cannot take into account the uncertainty of estimated parameters, which intrinsically exists in the clinical setting. Thus, we need to extend these control/optimization methods to robust ones. This paper is the first step towards such a direction by evaluating the worst case scenario among the bootstrap sample simulations. Expanding this line of research is a topic of the future research for theoreticians such as engineers, mathematical and/or computational biologists, physicists, mathematicians, and statisticians.

It is generally known that the bootstrap method is unreliable with a small sample dataset^36^. The main difference between what was discussed in^36^ and our method firstly described in^21^ is that we bootstrap time points of a time series and fit each bootstrapped time series to obtain a set of parameters. In addition, there are only a few patients classified to type (iii) originally^15,18^, which is consistent with our finding here, especially Table 1. Therefore, although the bootstrap method, in general, might be known to be unreliable for a small sample dataset, our method does not seem to inherit this weakness and can classify the three types without much problems, as shown in our previous paper^21^. As a result, the classifications obtained by the bootstrap method are correlated well with the classifications by simply fitting the whole dataset for each patient in^18^ (Table 3).

**Table 3:** Comparison of the classifications by the proposed method with the classifications in^18^ using the 46 Canadian patients.

ref.^18^ \Proposed bootstrap	Type (i)	Type (ii)	Type (iii)	Total
Type (i)	11 (58%)	8 (42%)	0 (0%)	19 (100%)
Type (ii)	9 (35%)	17 (65%)	0 (0%)	26 (100%)
Type (iii)	0 (0%)	0 (0%)	1 (100%)	1 (100%)
Total	20 (43%)	25 (54%)	1 (2%)	46 (100%)

With the current fitting approach, we need the first two and half cycles of PSA measurements for confidently estimating a set of parameters for the model^15,16,18^. Although the constraints on the parameters may cause some bias on their estimates, the constraints are necessary for fitting the data and reproducing the relapse^15^. By using our recent developments^26,37^ on techniques of machine learning and the Bayesian theorem, we may be able to estimate a set of parameter values based on much shorter PSA time series. This is another topic for our future research among the theoreticians.

Usually, in a hormone treatment for prostate cancer, some medicine is injected under the skin and its effect lasts for one month or three, depending on the kind and amount of the medicine. We assume here for the sake of simplicity that pharmacokinetics is not so problematic in our setting.

There is a limitation in our study. As shown in Table 2, there were only two patients in type (iii). Thus, in this manuscript, we tried to make conclusions by grouping type (ii) and type (iii). To derive stronger statements, we need to analyze a larger dataset such as ones in^13,14^.

We conclude that the mathematical model of prostate cancer of^15^ is useful for predicting behavior of PSA and hypothesize that use of the model could personalize the treatment schedule even if observations of the PSA levels obtained from a patient are noisy and short compared with time series obtained in the other fields. We overcame short noisy measurements of the biomarker by representing the uncertainties by sets of parameters and initial conditions obtained by 100 bootstrap samples according to the method of^21^ for each patient. IAS is the treatment of choice if the PSA pattern falls into type (i). We believe that the proposed approach could promote the introduction of mathematical models and tools to medical practice for robust treatment scheduling. This mathematical framework is quite general and one can apply the framework to other diseases once we construct their mathematical models and obtain short and noisy observations of some good biomarkers related to the diseases.

### Appendix A: Equations and Assumptions

#### A mathematical model for intermittent androgen suppression

This work is based on a mathematical model of prostate cancer under intermittent androgen suppression proposed in^15^. In this mathematical model, we assume that there are three kinds of cancer cells: one kind of androgen dependent cancer cells and two kinds of castration resistant cancer cells (see Fig. 1). The first kind of castration resistant cells is assumed to be generated through reversible changes such as adaptations, while the second kind of castration resistant cells is assumed to be generated through irreversible changes such as mutations. We let *x*_1_, *x*_2_, and *x*_3_ correspond to the androgen dependent cancer cells, the first kind of castration resistant cancer cells, and the second kind of castration resistant cancer cells, respectively, so that *x*_1_ + *x*_2_ + *x*_3_ represents simply the serum level of prostate specific antigen (PSA) in ng/mol. While the hormone therapy is prescribed, the tumor dynamics is assumed to follow the following dynamics:

1 $$\frac{d}{dt}(\begin{array}{c}{x}_{1}\\ {x}_{2}\\ {x}_{3}\end{array})=(\begin{array}{ccc}{w}_{1,1}^{1} & 0 & 0\\ {w}_{2,1}^{1} & {w}_{2,2}^{1} & 0\\ {w}_{3,1}^{1} & {w}_{3,2}^{1} & {w}_{3,3}^{1}\end{array})(\begin{array}{c}{x}_{1}\\ {x}_{2}\\ {x}_{3}\end{array})\equiv {W}_{1}(\begin{array}{c}{x}_{1}\\ {x}_{2}\\ {x}_{3}\end{array}).$$

While the hormone therapy is stopped, the tumor dynamics is assumed to follow the following dynamics:

2 $$\frac{d}{dt}(\begin{array}{c}{x}_{1}\\ {x}_{2}\\ {x}_{3}\end{array})=(\begin{array}{ccc}{w}_{1,1}^{0} & {w}_{1,2}^{0} & 0\\ 0 & {w}_{2,2}^{0} & 0\\ 0 & 0 & {w}_{3,3}^{0}\end{array})(\begin{array}{c}{x}_{1}\\ {x}_{2}\\ {x}_{3}\end{array})\equiv {W}_{0}(\begin{array}{c}{x}_{1}\\ {x}_{2}\\ {x}_{3}\end{array}).$$

For our fittings, we discretize the models in time by using the Euler approximation with the time resolution of a day, and obtain the following difference equations:

3 $$(\begin{array}{c}{x}_{1}(t+1)\\ {x}_{2}(t+1)\\ {x}_{3}(t+1)\end{array})=(\begin{array}{ccc}{d}_{1,1}^{1} & 0 & 0\\ {d}_{2,1}^{1} & {d}_{2,2}^{1} & 0\\ {d}_{3,1}^{1} & {d}_{3,2}^{1} & {d}_{3,3}^{1}\end{array})(\begin{array}{c}{x}_{1}(t)\\ {x}_{2}(t)\\ {x}_{3}(t)\end{array}),$$

for the on-treatment period, and

4 $$(\begin{array}{c}{x}_{1}(t+1)\\ {x}_{2}(t+1)\\ {x}_{3}(t+1)\end{array})=(\begin{array}{ccc}{d}_{1,1}^{0} & {d}_{1,2}^{0} & 0\\ 0 & {d}_{2,2}^{0} & 0\\ 0 & 0 & {d}_{3,3}^{0}\end{array})(\begin{array}{c}{x}_{1}(t)\\ {x}_{2}(t)\\ {x}_{3}(t)\end{array}),$$

for the off-treatment period. Here, ${w}_{i,j}^{m}$ and ${d}_{i,j}^{m}$ are related as ${w}_{i,j}^{m}={d}_{i,j}^{m}$ if $i\ne j$ and ${w}_{i,i}^{m}+1={d}_{i,i}^{m}$. As demonstrated in^15,18^, this set of mathematical models can be fitted into the clinical datasets of PSA in good accuracy.

#### Fitting resampled data

The additional assumption in this paper is that we resample the original dataset of PSA for each patient 100 times by following^21^, and then fit each of the resampled datasets by using the method of^15^ to obtain a set of parameters and initial conditions. Namely, the cost function can be written as

5 $$[\begin{array}{c}\frac{1}{K+{\varepsilon }_{1}}\{\sum _{k=1}^{K}|p({t}_{k})-\sum _{i=1}^{3}{\hat{x}}_{i}({t}_{k})|+{\varepsilon }_{2}\}\\ +\sum _{i,k}h({\hat{x}}_{i}({t}_{k}))\\ +\sum _{i,j,m}h({d}_{i,j}^{m})\\ +h({d}_{1,1}^{1}-0.8)+h(1.2-{d}_{1,1}^{1})+h({d}_{2,2}^{1}-0.8)+h(1.2-{d}_{2,2}^{1})+h({d}_{3,3}^{1}-0.8)\\ +h({d}_{3,3}^{1}-1)+h(1.2-{d}_{3,3}^{1})+h({d}_{1,1}^{0}-0.8)+h(1.2-{d}_{1,1}^{0})+h({d}_{2,2}^{0}-0.8)\\ +h(1.2-{d}_{2,2}^{0})+h({d}_{3,3}^{0}-0.8)+h(1.2-{d}_{3,3}^{0})+h({d}_{2,1}^{1})+h(0.1-{d}_{2,1}^{1})+h({d}_{3,1}^{1})\\ +h(0.1-{d}_{3,1}^{1})+h({d}_{3,2}^{1})+h(0.1-{d}_{3,2}^{1})+h({d}_{1,2}^{0})+h(0.1-{d}_{1,2}^{0})\\ +h(\sum _{i=1}^{3}{d}_{i,1}^{1}-0.8)+h(1.2-\sum _{i=1}^{3}{d}_{i,1}^{1})+h(\sum _{i=2}^{3}{d}_{i,2}^{1}-0.8)+h(1.2-\sum _{i=2}^{3}{d}_{i,2}^{1})\\ +h(\sum _{i=1}^{2}{d}_{i,2}^{0}-0.8)+h(1.2-\sum _{i=1}^{2}{d}_{i,2}^{0})\\ +h(2-\sum _{i=1}^{3}{\bar{x}}_{i}(360))+h(\sum _{i=1}^{3}{\bar{x}}_{i}(360\times 5)-10)\end{array}],$$

where *ε*_1_ = 0.000000001 and *ε*_2_ = 0.001, were newly introduced here to avoid dividing with 0. The PSA value at time *t*_*k*_ is denoted by *p*(*t*_*k*_). Variables $\hat{x}(t)$ and $\bar{x}(t)$ correspond the simulations at time *t* under IAS and CAS, respectively. The function *h*(*z*) is defined as

6 $$h(z)=\{\begin{array}{cc}100(1-z), & \mathrm{if}\,z < 0,\\ 0, & {\rm{o}}{\rm{t}}{\rm{h}}{\rm{e}}{\rm{r}}{\rm{w}}{\rm{i}}{\rm{s}}{\rm{e}},\end{array}$$

which ensures that the inside *z* of *h*(*z*) becomes positive at the end of the minimization process^15,38^. Therefore, as a whole, we try to obtain a set of parameters such that (i) the PSA values are non-negative, (ii) their temporal changes are constrained within finite ranges, and (iii) the cancer will relapse if we continue CAS for a long time. We minimized the cost function of equation (5) over $\{{d}_{i,j}^{m}\}$ and *x*(0) by the differential evolution algorithm^38^. Thus, we finally obtained 100 sets of parameters and initial conditions to represent the uncertainties due to the short and noisy measurements of PSA.

#### Classifications for patients

For each set of parameters for each patient, we applied the criteria of^15,18,35^, derived analytically, to classify each resampled dataset of each patient (see Fig. 3): If a parameter set belongs to type (i), namely if there exists a parameter *α* ∈ (0, 1) such that all the eigenvalues of *αW*_1_ + (1 − *α*)*W*_0_ are negative, the prostate cancer will be successfully suppressed by intermittent androgen suppression; If a parameter set belongs to type (ii), namely if the parameter set is not type (i) and (${w}_{1,1}^{1} > {w}_{1,1}^{0}$ or ${w}_{2,2}^{1} > {w}_{2,2}^{0}$ or ${w}_{3,3}^{1} > {w}_{3,3}^{0}$), the growth of prostate cancer will not be suppressed but will be delayed successfully longer by the intermittent androgen suppression than by the continuous androgen suppression; If a parameter set belongs to type (iii), namely if the parameter set is neither type (i) nor type (ii), the growth of the prostate cancer will be delayed by the continuous androgen suppression longer than the intermittent androgen suppression in any schedule. Because there are 100 parameter sets for each patient, we count the number of parameter sets for each type to obtain the most frequent type for each patient, which probably represents the patient’s tumor characteristic the best.

### Data availability statements

There are restrictions related to our data sharing because we used the clinical datasets, which are subject to patients’ privacy. Thus, we will share the datasets we used with interested readers after all the involving ethics committees including the readers’ approve the data sharing.

## Electronic supplementary material

Supplementary Information
