# Supplementary material for: Personalizing Androgen Suppression for Prostate Cancer Using Mathematical Modeling
Source: Sci Rep. 2018 Feb 8;8:2673. doi: 10.1038/s41598-018-20788-1 (PMC5805696; doi:10.1038/s41598-018-20788-1)
Supplement: Supplementary file 1 — Supplementary Information [file 41598_2018_20788_MOESM1_ESM.pdf]

Supplementary Information for Personalizing Androgen Suppression for Prostate Cancer Using Mathematical Modeling

Yoshito Hirata, Kai Morino, Koichiro Akakura, Celestia S. Higano, and Kazuyuki Aihara

Supplementary Figure 1
Supplementary Tables 1-3

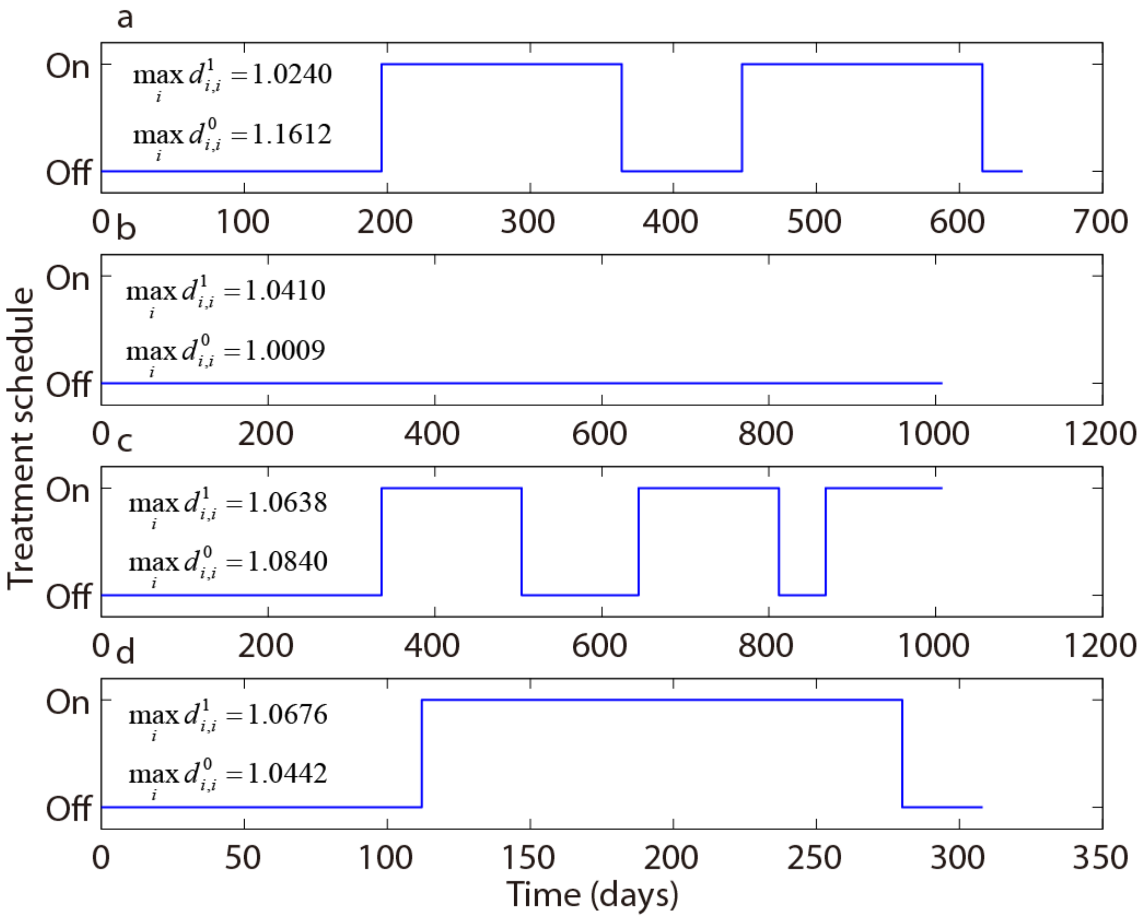

Supplementary Figure 1 | Examples for the optimal schedules for various type (i) patients. We also show the parameters for the maximal growth rates $\max_i d^1_{i,i}$ and $\max_i d^0_{i,i}$ among the cancer cells for the on-treatment period and the off-treatment period, respectively, so that we can explain why these particular schedules were chosen as the most robust. For example, in panel b, the schedule with the off-treatment for the whole period of 36 months was chosen because the maximal growth rate for the on-treatment period is much larger than that for the off-treatment period.

Supplementary Table 1: Statistics for the estimated parameters for a patient of type (i).

Parameters	Values (mean ± standard deviation)
$x_1(0)$	26.3899 ± 5.8061
$x_2(0)$	2.2925 ± 5.8135
$x_3(0)$	0.3177 ± 0.4876
$d_{1,1}^{1}$	0.9318 ± 0.0361
$d_{2,1}^{1}$	0.0095 ± 0.0138
$d_{2,2}^{1}$	0.9920 ± 0.0160
$d_{3,1}^{1}$	0.0003 ± 0.0009
$d_{3,2}^{1}$	0.0003 ± 0.0006
$d_{3,3}^{1}$	1.0034 ± 0.0054
$d_{1,1}^{0}$	0.9968 ± 0.0372
$d_{1,2}^{0}$	0.0548 ± 0.0382
$d_{2,2}^{0}$	1.0051 ± 0.0156
$d_{3,3}^{0}$	0.9722 ± 0.0586

Supplementary Table 2: Statistics for the estimated parameters for a patient of type (ii).

Parameters	Values (mean ± standard deviation)
$x_1(0)$	7.4102 ± 0.5975
$x_2(0)$	0.2194 ± 0.1740
$x_3(0)$	0.0000 ± 0.0000
$d^1_{1,1}$	0.9321 ± 0.0449
$d^1_{2,1}$	0.0042 ± 0.0072
$d^1_{2,2}$	0.9942 ± 0.0041
$d^1_{3,1}$	0.0000 ± 0.0000
$d^1_{3,2}$	0.0000 ± 0.0001
$d^1_{3,3}$	1.0248 ± 0.0156
$d^0_{1,1}$	0.9998 ± 0.0008
$d^0_{1,2}$	0.0999 ± 0.0007
$d^0_{2,2}$	0.9999 ± 0.0009
$d^0_{3,3}$	0.9306 ± 0.0916

Supplementary Table 3: Statistics for the estimated parameters for a patient of type (iii).

Parameters	Values (mean ± standard deviation)
$x_1(0)$	8.7560 ± 1.7386
$x_2(0)$	1.9175 ± 1.7583
$x_3(0)$	0.0265 ± 0.1245
$d_{1,1}^{1}$	0.8605 ± 0.0569
$d_{2,1}^{1}$	0.0221 ± 0.0231
$d_{2,2}^{1}$	0.9946 ± 0.0192
$d_{3,1}^{1}$	0.0008 ± 0.0025
$d_{3,2}^{1}$	0.0001 ± 0.0004
$d_{3,3}^{1}$	1.0057 ± 0.0050
$d_{1,1}^{0}$	0.9842 ± 0.0343
$d_{1,2}^{0}$	0.0665 ± 0.0339
$d_{2,2}^{0}$	0.9903 ± 0.0414
$d_{3,3}^{0}$	0.9944 ± 0.0600
